# Perspectives Toward Damage‐Tolerant Nanostructure Ceramics

**DOI:** 10.1002/advs.202309834

**Published:** 2024-04-06

**Authors:** Meicen Fan, Tongqi Wen, Shile Chen, Yanhao Dong, Chang‐An Wang

**Affiliations:** ^1^ State Key Lab of New Ceramics and Fine Processing School of Materials Science and Engineering Tsinghua University Beijing 100084 China; ^2^ Department of Mechanical Engineering The University of Hong Kong Hong Kong SAR China

**Keywords:** ceramics, damage tolerance, fibrous aerogels, nanostructures, machine learning

## Abstract

Advanced ceramic materials and devices call for better reliability and damage tolerance. In addition to their strong bonding nature, there are examples demonstrating superior mechanical properties of nanostructure ceramics, such as damage‐tolerant ceramic aerogels that can withstand high deformation without cracking and local plasticity in dense nanocrystalline ceramics. The recent progresses shall be reviewed in this perspective article. Three topics including highly elastic nano‐fibrous ceramic aerogels, load‐bearing nanoceramics with improved mechanical properties, and implementing machine learning‐assisted simulations toolbox in understanding the relationship among structure, deformation mechanisms, and microstructure‐properties shall be discussed. It is hoped that the perspectives present here can help the discovery, synthesis, and processing of future structural ceramic materials that are insensitive to processing flaws and local damages in service.

## Graphical abstract

Selective topics of damage‐tolerant nanostructure ceramics, including elastic nano‐fibrous ceramic aerogels, load bearing nanoceramics, machine learning assisted simulations of microstructure‐properties relationship, are revived in the present manuscript. The perspectives present here can help the discovery, synthesis, and processing of future structural ceramics.

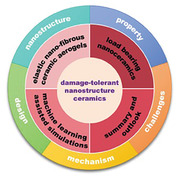

## 1 Introduction

The intrinsic brittleness of ceramic materials poses a significant obstacle to their widespread practical applications.^[^ ^1^, ^2^ ^]^ The formidable chemical bonding, comprising ionic and covalent bonds, and the rigidly ordered crystalline structure, lacking a sliding mechanism, contribute to the characteristic high hardness and brittleness of ceramics. Consequently, these materials are susceptible to fractures under external impact or stress. To surmount this challenge, researchers continually explore novel nanostructure designs and material preparation methods aimed at achieving damage‐tolerant ceramics, enhancing their mechanical properties, and broadening their range of applications.^[^ ^3^, ^4^ ^]^ Furthermore, the ongoing advancements in machine learning and simulation technology offer a fresh perspective for the investigations of nanoceramics.

In the last two decades, nanoceramics have emerged as a novel category of structural ceramic materials, distinguishing themselves from traditional ceramics.^[^ ^5^, ^6^ ^]^ Characterized by nano‐scale structures, including grain size, multiple phases, and flaws all below 100 nm, nanoceramics exhibit significant advancements in mechanical properties, high‐temperature resistance, and machinability. With continuous developments in advanced ceramic synthesis and characterization technologies, nanoceramics have progressively assumed a crucial role in diverse fields such as structural parts, electronics, and energy.^[^ ^7^, ^8^, ^9^ ^]^ This review is focused on damage‐tolerant ceramic nanostructures, highlighting recent progress in enhancing the mechanical properties of nanoceramics. Notable strategies include elastic nano‐fibrous ceramic aerogels and load‐bearing nanoceramics. The elastic structure design of nano‐fibrous ceramic aerogels involves enhancing the movement flexibility of individual nanofibers, designing connecting nodes between nanofibers, and incorporating additional pore structures. On the other hand, load‐bearing nanoceramics address challenges like the density‐strength trade‐off in porous ceramics, microstructure design in dense nanocrystalline ceramics, ceramic matrix composites, and directional structural design. Additionally, this review discusses the latest advancements in machine learning‐assisted simulation and the application of various simulation methods in the field of nanoceramics.

## 2 Elastic Nano‐Fibrous Ceramic Aerogels

Since the initial discovery of silica aerogels in 1931, ceramic aerogels have garnered significant attention, owing to their outstanding properties, including high porosity, lightweight nature, and exceptional heat insulation. A series of elastic ceramic aerogels have been reported one after another inspired by the development strategy of elastic carbon tube aerogels, and the preparation of nano‐fibrous ceramic aerogels has become one of the most important ways to overcome the intrinsic brittleness of ceramics and conventional brittle silica aerogels through microstructural design.^[^ ^10^ ^]^ This family of elastic ceramic aerogels is witnessing continuous expansion, evolving year by year, and progressively branching into the realm of multifunctionality.^[^ ^11^, ^12^, ^13^, ^14^ ^]^

The elasticity inherent in ceramic aerogels primarily arises from the remarkable flexibility of individual nanofibers and the interconnected nodes formed by the overlapping nanofibers. Additionally, the porous structure, formed through the intricate winding of numerous nanofibers, contributes to the material's deformation capability. Throughout the deformation process, the elastic buckling and movement of each nanofiber play a pivotal role in providing deformation, while the porous structure affords additional freedom of movement for the nanofibers. Simultaneously, the nodes between adjacent nanofibers facilitate the reversible compression of the aerogels by constraining nanofiber distortion, imparting stiffness, strength, and modulus to the material. Consequently, the design of the elastic structure in nanofibrous ceramic aerogels involves three key considerations: enhancing the flexibility of individual nanofibers, designing effective connection nodes between fibers, and incorporating additional pore structures.

Generally speaking, synthesis methods of fibrous ceramic aerogels include electrospinning, chemical vapor deposition, freeze‐drying, and template‐assisted method. The characteristics of resilient ceramic aerogels heavily rely on the varied preparation methods employed, resulting in distinct microstructures. For instance, aerogels produced through electrospinning and chemical vapor deposition commonly manifest a randomly distributed microstructure, while the directional freeze‐drying technique is adept at crafting anisotropic aerogels. Furthermore, the template‐assisted method typically results in the formation of periodic and hyperbolic structures.

The advancement of elastic nanofibrous ceramic aerogels, endowed with a harmonious blend of mechanical resilience and functional versatility, greatly amplifies their applicability across diverse industries. Notably, the appeal of lightweight and robust fibrous ceramic aerogels extends to aerospace applications, encompassing vital roles in structural components, thermal protection systems, and spacecraft insulation. Leveraging their high surface area and porosity, fibrous ceramic aerogels prove efficacious in catalysis, noise absorption, and water purification. The integration of functional components further augments their utility in electronics, sensors, and energy storage devices like supercapacitors and flexible wearable electronics. The thermal insulation prowess exhibited by fibrous ceramic aerogels, owing to their low densities, nanoporous structures, and radiation absorption elements, not only enhances energy efficiency but also curtails heat transfer. This multifaceted approach encompasses a reduction in conduction in solids, as well as mitigated conduction and convection in air, and the retardation of thermal radiation. **Table** **1** summarizes properties of elastic nano fibrous ceramic aerogels reported in the literature.

**Table 1:** Summarized properties of elastic nano fibrous ceramic aerogels reported in the literature. Notation: *ρ* for density, *E* for modulus, *κ* for thermal conductivity, *T*_max_ for maximum service temperature, *α* for thermal expansion coefficient, *ν* for Poisson's ratio.

Materials	*ρ* [mg cm^−3^]	*E* [kPa]	*κ* [mW m^−1^ K^−1^]	*T*_max_ [°C]	*α* [K^−1^]	*ν*	References
SiC‐SiO*_x_*	5.7	204	28.4	1200	/	1.98	[15]
ZrO_2_	20	73 500	104	1300	1.2 × 10^−7^	3.3 × 10^−4^	[18]
Mullite	2.15	62 000	26.2	1500	/	0.15	[19]
Mullite	6	2.9	22.8	1400	/	/	[20]
SiC@SiO_2_	6.5	160.8	14	1200	/	−0.08	[21]
Si‐Al	10	/	29	1300	/	/	[23]
SiO_2_	0.5	9	22.3	1100	/	/	[24]
SiC@PyC	40	/	/	1400	/	/	[25]
SiO_2_	0.25	7	24	1100	/	/	[26]
SiO_2_	0.1	25	20	1400	−1.8 × 10^−6^	−0.25	[27]
Carbon nanotube	0.008	/	23	/	/	/	[28]

### 2.1 Increase the Motion Flexibility of a Single Nanofiber

Enhancing the flexibility of individual ceramic nanofibers represents a pivotal solution to addressing the mechanical properties of ceramic aerogels. Augmenting the kinematic flexibility of ceramic nanofibers facilitates easier deformation under external forces, enabling the material to bend and stretch more extensively, thereby enhancing its overall deformability. Furthermore, flexible nanofibers play a crucial role in dispersing and absorbing energy during external stresses. This capacity enables the material to effectively buffer impact and strain, slowing the rate of crack propagation.

**Figure 1:**
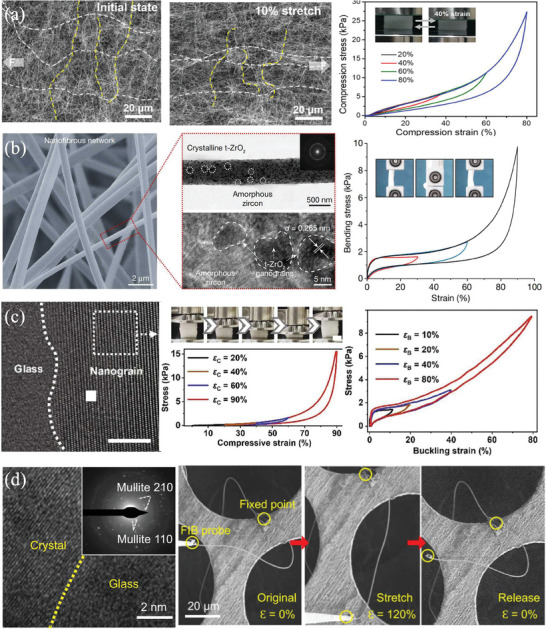
Increase the motion flexibility of single nanofiber. a) Crimped SiC‐SiO*x* twin‐crystal nanofiber. Reproduced with permission.^[^ ^15^ ^]^ Copyright 2021, American Chemical Society. b) Hypocrystal ceramics aerogels with “double zero” abnormal physical properties. Reproduced with permission.^[^ ^18^ ^]^ Copyright 2022, Springer Nature. c) Nanocrystals in amorphous matrix. Reproduced with permission.^[^ ^19^ ^]^ Copyright 2022, Elsevier. d) Fine mullite particles and glass phases dual‐phase ceramics aerogels. Reproduced with permission.^[^ ^20^ ^]^ Copyright 2022, Springer Nature.

Curved nanofibers that exhibit enhanced deformability stands out as an effective strategy for improving the flexibility of individual ceramic nanofibers. As exemplified by Wang et al., the utilization of crimped SiC‐SiOx twin‐crystal nanofibers demonstrated noteworthy outcomes. The inherent curvature of these nanofibers facilitated deformation, interaction, and reorientation during stretching, effectively mitigating stress concentration. This, in turn, hindered both the initiation and propagation of cracks, resulting in commendable tensile properties. Such an approach has successfully yielded highly compressible ceramic aerogels with reversible stretching capabilities and remarkable resistance to crack formation (**Figure** **1**a).^[^ ^15^ ^]^

Integrating composite structures of amorphous and crystal materials in nanoceramics represents an alternative approach to augmenting the motility flexibility of individual ceramic nanofibers. Achieving a balanced and synergistic effect between amorphous and crystal components is a crucial strategy for attaining a harmonious blend of strength and elasticity.^[^ ^16^ ^]^ The periodic arrangement of crystal materials imparts higher strength but lower ductility to the ordered structure. Conversely, amorphous materials lack long‐term periodicity in their atomic and molecular arrangements. The extensive variation range of bond length and bond angle, along with the restorability of lattice spacing changes in amorphous materials, plays a pivotal role in facilitating relaxation and the release of deformation, ultimately contributing to superior elasticity. Wang et al. elucidate that the micro‐plasticity of amorphous materials is achieved through the flipping events of highly distorted coheedral tetrahedra within the Delaunay network, akin to dislocations in crystals.^[^ ^17^ ^]^ Duan et al. used the zig‐zag structure design of hypocrystal ceramics to give the ceramic aerogels abnormal physical properties of “double zero” with near zero Poisson ratio (3.3 × 10^−4^) and near zero thermal expansion coefficient (1.2 × 10^−7^ K^−1^), and the elastic recovery compressive strain of the material was as high as 95%. Both excellent tensile (fracture strain >40%) and bending deformation capacity (bending strain >90%) were attained with almost zero strength loss and volume shrinkage during 10 000 high‐frequency severe thermal shock (≈200 °C s^−1^) and long‐term high temperature (>1000 °C) aerobic exposure. In addition, semi‐crystal ceramics show a stronger coating ability on carbon, improve the high‐temperature oxidation resistance of carbon materials, so as to effectively block the high‐temperature thermal radiation, and achieve the lowest high‐temperature thermal conductivity of porous low‐density ceramic aerogel (20 mg cm^−3^, less than 104 mW mK at 1000 °C) (Figure 1b). It makes up for the shortcomings of lightweight aerogels in the field of high‐temperature thermal insulation.^[^ ^18^ ^]^ Wu et al. embedded nanocrystals into an amorphous matrix to prevent the sliding of nanocrystal domains and used nanocrystal domains to limit the migration of amorphous matrix at high temperatures, with excellent thermomechanical properties from −196 °C to 1500 °C. In this sub‐crystal design, the deformation of ceramic fibers under mechanical and thermal excitation will exist in a high‐order buckling mode instead of a uniform mode, thus providing additional degrees of freedom to promote the high‐order deformation mode, that is, full recovery under large deformation of more than 90% compression strain or 80% buckling strain at a Poisson's ratio of close to zero, with no cumulative damage or structural collapse during 10 000 large‐scale compression or buckling cycles (Figure 1c).^[^ ^19^ ^]^ Ding et al. synthesized ceramic nanofibers composed of fine mullite particles of many grain boundaries and glass phases by electrospinning. The material can be stretched from its original form to 100% tensile strain without breaking, while showing excellent resilience under large deformation exceeding 60% compressive strain or 90% buckling strain, as well as good fatigue resistance up to 100 000 cycles. In addition, it has thermal stability from −196 °C to 1400 °C, repeatable stretchability at 1300 °C, and low thermal conductivity of 0.0228 W m^−1^ K^−1^ in air (Figure 1d).^[^ ^20^ ^]^

### 2.2 Design Connecting Nodes of Nano‐Fibrous Aerogels

In nano‐fibrous aerogels, the “connecting nodes” pertain to the structures formed at points where the fibers intersect. The exceptional crimp properties observed in 2D ceramic aerogels and the impressive compression‐rebound ability exhibited by 3D aerogel sponges can be attributed to the stabilizing influence of these connecting nodes. The effectiveness of these nodes plays a pivotal role in determining whether an aerogel can maintain its structural integrity following deformations. Furthermore, the fixation effect of connecting nodes proves crucial in dispersing stress during external forces, thereby impeding crack propagation and enhancing the overall toughness of the aerogels. As a result, a key focus in optimizing elastic nano‐fibrous aerogels revolves around refining the design of connecting nodes. This involves reinforcing the binding force between inherent connecting nodes and introducing additional connecting nodes to further bolster the aerogel's structural resilience.

**Figure 2:**
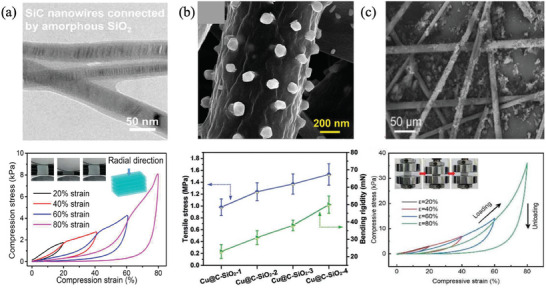
Strengthen the binding force between the inherent connecting nodes in ceramic aerogels. a) SiC nanofibers welded by SiO_2._ Reproduced with permission.^[^ ^21^ ^]^ Copyright 2020, The American Association for the Advancement of Science. b) Modify the morphology of SiO_2_ fibers by Cu nanoparticles. Reproduced with permission.^[^ ^22^ ^]^ Copyright 2019, The Royal Society of Chemistry. c) Bird nest composite ceramic aerogels. Reproduced with permission.^[^ ^23^ ^]^ Copyright 2023, John Wiley and Sons.

By strategically designing the composition of connecting nodes or enhancing fiber roughness, along with devising improved intersectional structures such as a bird nest configuration, it becomes feasible to augment the bonding force when fibers naturally intersect. Pan et al. sintered a layer of amorphous SiO_2_ on the surface of SiC nanofibers and prepared anisotropic SiC@SiO_2_ honeycomb aerogel by directional freeze casting (**Figure** **2**a). The adjacent SiC nanofibers were welded by oxides, and the binding force was enhanced, which made the aerogel recover its original appearance after compressive strain.^[^ ^21^ ^]^ Ding et al. introduced Cu nanoparticles to modify the morphology of SiO_2_ fibers (Figure 2b), increase the friction between fibers, and offset the external stress, so as to improve the mechanical properties of the fiber.^[^ ^22^ ^]^ Wang et al prepared a flexible silica‐alumina bird nest composite ceramic aerogels by the macro‐molecular template sacrifice method (Figure 2c). The resulting cross‐interlocking reinforced structure, akin to a bird nest configuration, is entirely composed of ceramic fiber lapping. Despite the absence of any chemical reactions, this structure imparts significant strength and excellent compressive resilience to the aerogel, allowing it to fully rebound even under an 80% compressive strain.^[^ ^23^ ^]^

**Figure 3:**
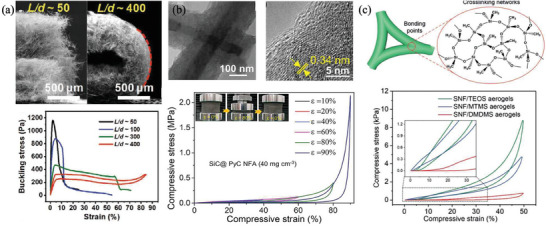
Bring in additional connecting nodes. a) Increased L/d ratio of nanofibers enhances the flexural elasticity. Reproduced with permission.^[^ ^24^ ^]^ Copyright 2020, John Wiley and Sons. b) PyC nodes convert the movable connected nodes between SiC nanofibers into fixed connected nodes. Reproduced with permission.^[^ ^25^ ^]^ Copyright 2022, Elsevier. c) Three crosslinkers in a “rubber‐like” elastic bonding network. Reproduced with permission. Reproduced with permission.^[^ ^26^ ^]^ Copyright 2020, John Wiley and Sons.

The bending and compression‐rebound capabilities of nano‐fibrous aerogels can undergo substantial enhancement through the introduction of additional connecting nodes achieved by manipulating the length/diameter ratio (L/d ratio) of fibers or by judiciously selecting suitable crosslinkers. For instance, Ding et al. observed that interwoven structures formed by fibers with an elevated L/d ratio contribute significantly to the flexural elasticity of fiber sponges. Conversely, a reduced L/d ratio in individual fibers results in noticeable brittleness, a sharp decline in stress, and immediate cracking of the porous structure. In the case of SiO_2_ fiber sponges, a single fiber with an L/d ratio surpassing 400 facilitates easier bending and rebounding (**Figure** **3**a).^[^ ^24^ ^]^ Wang et al. used the good bonding effect of PyC nodes between SiC nanofibers to convert the movably connected nodes between adjacent nanofibers into fixed connected nodes. This alteration led to an increased node count in the aerogel structure, resulting in a substantial boost in Young's modulus and compressive stress, 60 and 36 times higher, respectively than the control SiC nanofiber aerogel (Figure 3b).^[^ ^25^ ^]^ Additionally, Ding et al. utilizing silicon oxide nanofibers as the foundational element, opted for three crosslinkers to construct an in situ “rubber‐like” elastic bonding network between fibers. This innovative approach allowed for the creation of a super‐elastic silica nanofiber aerogel with a frame structure, capable of recovering under 90% compressive strain (Figure 3c).^[^ ^26^ ^]^

### 2.3 Additional Pore Structures

**Figure 4:**
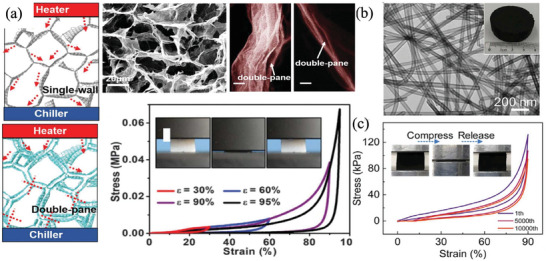
Bring in additional pore structures in the nanoceramic materials. a) Double‐wall porous structure BN ceramic aerogels. Reproduced with permission.^[^ ^27^ ^]^ Copyright 2019, The American Association for the Advancement of Science. b,c) Hollow carbon tube aerogels. Reproduced with permission.^[^ ^28^ ^]^ Copyright 2019, Elsevier.

Furthermore, introducing additional pore structures into nanoceramic materials proves to be a pivotal strategy in enhancing the capacity to accommodate deformation. Duan et al. achieved noteworthy success in fabricating hyperbolic double‐wall porous structure BN ceramic aerogels, characterized by a negative Poisson ratio and negative thermal expansion coefficient (**Figure** **4**a). This specially designed double‐layer aerogel can not only reduce the wall thickness due to its double‐layer pane wall but also reduce the stiffness of the aerogel and enhance the toughness of the material due to the multi‐void structure. More importantly, the double‐layer structure and porous structure provide additional freedom to release deformation, and reduce the mutual constraints between the structural elements, 95% of the compressive strain can be restored to the original shape. In addition, the zigzag thermal channels in the double‐layer structure inhibit air conduction and convection, reducing the solid conductive contribution, and resulting in ultra‐low thermal conductivity.^[^ ^27^ ^]^ Inspired by the hollow structure of polar bear hair, Yu et al. prepared hollow carbon tube aerogels that can maintain structural integrity after more than 1 million cycles at 30% strain and 10 000 cycles of compression and release at 90% strain (Figure 4b,c).^[^ ^28^ ^]^

## 3 Load Bearing Nanoceramics

Although nanoceramic aerogels exhibit commendable elasticity under external stress, their strength and load‐bearing capacity, attributed to their porous structure and low density, demand further enhancement to effectively address stress‐induced damage. Broadly speaking, numerous reinforcement strategies for nanoceramics have been put forth. These encompass addressing the density‐strength trade‐off in porous ceramics, refining microstructure design in dense nanocrystalline ceramics, incorporating reinforced ceramic matrix composites, and implementing directional structural design. Each of these approaches aims to fortify the mechanical properties of nanoceramic aerogels, thereby ensuring heightened resilience and durability in the face of stress challenges.

### 3.1 Resolving Density‐Strength Trade‐off in Porous Ceramics

High strength often requires high matrix density and low porosity, which is contrary to the requirements of flexible porous structures. Therefore, with the development of nanoceramics, it is vital to resolving density‐strength trade‐off in porous ceramic and obtain high mechanical properties while maintaining low densities and desirable properties. Simply speaking, the design rules can be summarized as optimizing the bonding of connection in porous ceramic, homogenizing the size and shape of pore structure, and adjusting the distribution of pore structure, such as designing hierarchical porous structure and hollow pore structure. **Table** **2** summarizes the preparation methods of porous ceramics.

**Table 2:** Summary of the preparation method of porous ceramics.

	Preparation method	Pore size	Porosity [%]	Advantages	Disadvantages
Pore formers	Removing the pore formers at a high temperature	10 µm–1 mm	0–50	Controllable pore size and shape	Poor distribution, low porosity
Organic foam impregnation	Removing organic foam at a high temperature	100 µm–5 mm	70–90	Controllable pore size, high porosity, high strength	Uncontrollable product shape, not environmentally friendly
Foaming process	Add foaming agent and foam into pores in the suspension	10 µm–2 mm	40–90	Closed pores, high porosity, high strength	High raw material requirements, difficult preparation process
Freeze‐drying process	Frozen ceramic paste is sublimed directly by reducing the pressure	10 µm–200 µm	30–85	High orientation, high porosity	High equipment requirements, high cost, low production efficiency
Particle stacking process	By coarse aggregate bonding	0.1 µm–600 µm	<50	Simple process, high strength	Low porosity
Sol‐gel method	The sol formed a spatial network in gel process	2 nm–100 mm	0–95	Uniform pore distribution, nano pores	Low yields, limited raw materials, and product shapes
3D printing	Layer‐by‐layer superposition molding	50 µm–500 µm	40–80	Controllable pore size and shape	High equipment requirements, difficult preparation process

**Figure 5:**
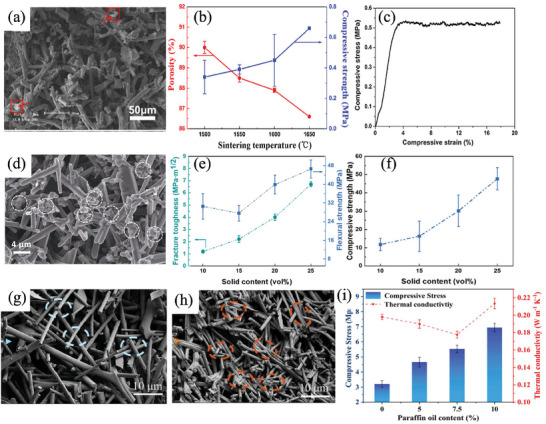
Optimizing the bonding of connection for porous fibrous ceramic to attain high strength. a) Microstructure of YSZ fibers with K_2_SO_4_ sintering aid addition at 1500 °C. b) Variation of porosity and compressive strength of fibrous YSZ ceramics at different sintering temperature and c) Compressive strain‐stress curve of the specimens sintered at 1650 °C. Reproduced with permission.^[^ ^29^ ^]^ Copyright 2012, Elsevier. d) Reinforced porous Si_3_N_4_ fiber ceramics by strong SiC second phase distributed in the lap of β‐Si_3_N_4_ and its e) Fracture toughness and flexural strength. f) Compressive strength. Reproduced with permission.^[^ ^30^ ^]^ Copyright 2021, Springer Nature. g) Solid mullite fibers‐based ceramics. h) Porous mullite fiber‐based ceramics and i) Their mechanical and thermal properties with different porous structures. Reproduced with permission.^[^ ^31^ ^]^ Copyright 2022, Elsevier.

Enhancing the bonding between connections proves to be a highly effective strategy for achieving elevated strength in porous ceramics. The strength of the bonds formed among interconnected ceramic fibers is a pivotal factor influencing the overall strength of the porous ceramic. Robust bonding not only reinforces the structural integrity but also facilitates more efficient stress dispersion under external forces. Dong found that K_2_SO_4_ provides a liquid phase above its melting point at the junctions of YSZ fibers, which promotes the diffusion mass transfer and bonding of the YSZ fibers together, while K_2_SO_4_ will be cleared away by evaporation holding at a higher temperature. In comparison, YSZ fibers without K_2_SO_4_ addition showed cracks and almost no mechanical strength (**Figure** **5**a–c).^[^ ^29^ ^]^ Chen et al. prepared reinforced porous Si_3_N_4_ fiber ceramics by strong SiC second phase distributed in the lap of *β*‐Si_3_N_4_ which plays a role of napping grain boundaries, showing much higher strength than that values in literature (Figure 5d–f).^[^ ^30^ ^]^ Guo et al. reported a strategy of using porous mullite fibers (Figure 5h) instead of solid mullite fibers (Figure 5g) to fabricate porous mullite fiber‐based ceramics, both of them exhibited similar bulk density (≈0.8 g cm^−3^) which means porous mullite fibers were much denser resulting in an increase in the number of bond points between fibers than solid mullite fibers. Therefore, porous mullite fiber‐based ceramics exhibited a higher compressive strength (5.53 MPa) than solid mullite fiber‐based ceramics (3.21 MPa) (Figure 5i).^[^ ^31^ ^]^

**Figure 6:**
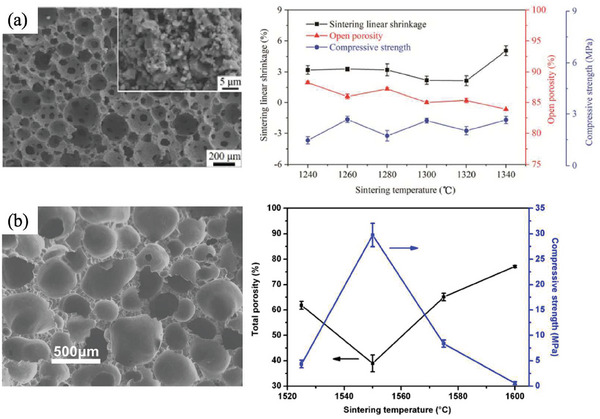
Pore structure homogenization. a) SEM images of high equability of the spherical pores in cordierite and its sintering shrinkage, open porosity, and compressive strength. Reproduced with permission.^[^ ^32^ ^]^ Copyright 2022, Springer Nature. b) Porous silica ceramics with uniform pores and their total porosity and compressive strength. Reproduced with permission.^[^ ^33^ ^]^ Copyright 2016, Elsevier.

Homogenizing the size and shape of the pore structure can lead to a uniform structure, which makes the stress distribution uniform and reduces the stress concentration under external force. Qiao and Wang et al. prepared the porous cordierite by the in situ solid‐state reactions, which showed a compressive strength of 1.51–2.65 MPa with a porosity of 83.9–87.8%. Herein, the high strength was caused by the high equability of the spherical pores and the small sintering deformation (**Figure** **6**a).^[^ ^32^ ^]^ Yang et al. obtained porous silica ceramics with uniform pores through direct sintering densely packed silica poly‐hollow microspheres which could in situ foam to form homogeneous pores in the inert atmosphere. The porous silica ceramics with a porosity of 39.0–77.2% showed compressive strength ranging from 0.67 ± 0.28 MPa to 29.77 ± 2.28 MPa (Figure 6b).^[^ ^33^ ^]^

**Figure 7:**
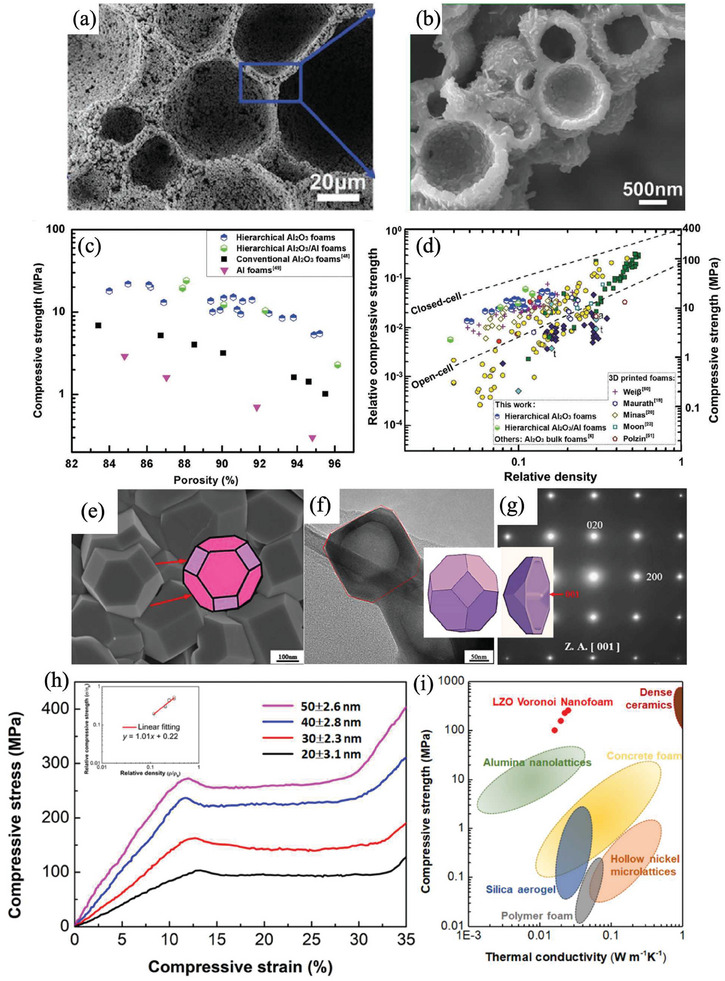
Pore structure optimizing. a,b) Microstructures of the hierarchical porous materials and c,d) Its mechanical properties in comparison to literature. Reproduced with permission.^[^ ^35^ ^]^ Copyright 2020, John Wiley and Sons. e–g) Decahedral La_2_Zr_2_O_7_ with approximately spherical inner hollow and h,i) Its excellent mechanical performance. Reproduced with permission.^[^ ^36^ ^]^ Copyright 2021, Elsevier.

Adjusting the distribution of pores, such as designing hierarchical porous structures and hollow pore structures can also obtain high‐strength porous ceramics. The dependence of the mechanical properties on the relative density for porous materials can be described by the Ashby model^[^ ^34^ ^]^:

(1) $$\frac{\sigma_{f}}{\sigma_{f,b}}=\;\beta {\left(\frac{\rho }{\rho_{b}}\right)}^{m\;}$$

where *σ_f_* and *σ*_f,b_ are the fracture strength of the porous materials and corresponding dense materials respectively. *ρ* and *ρ*_f,b_ are the density of the porous materials and corresponding bulk materials respectively. The constants *β* and *m* depend on the structure of the cellular solid, with *m *= 3/2 and 1 for ideal open‐cell and closed‐cell foams, respectively. The compressive strength of the porous foams typically lies within the values expected for these two idealized scenarios. Yang et al. generated hierarchical porous Al_2_O_3_ composites via direct foaming and thermal oxidation of aluminum particles with an unprecedented compressive strength of 14.8 and 5.3 MPa at porosity levels as high as 90% and 95%, respectively, which are 7–8 times higher than conventional counterparts with pores at a single length scale. This is because the hierarchical structure had a more uniform stress distribution, thus preventing extensive distortion and the local stress concentration. In the work of ref. [35], *m *= 1.06 was obtained which is close to 1 predicted for ideal closed‐cell structures (**Figure** **7**a–d).^[^ ^35^ ^]^ Besides, using highly dispersed carbon spheres as a template, Li et al. synthesized decahedral La_2_Zr_2_O_7_ with an approximately spherical inner hollow by a two‐step sintering process which showed very high strength and superior thermal superinsulation up to 1400 °C. The approach of making these nanoporous ceramics in bulk form is very general and can be applied to a wide variety of ceramics besides La_2_Zr_2_O_7_. The prepared centimeter‐size sample with 69.1% porosity has an ultra‐high compressive strength of 259 MPa (while the value for dense La_2_Zr_2_O_7_ is 518 MPa) and a high bending strength of 100 MPa. Such high values result from the Voronoi porous structure with tightly packed tetrahedral grains, unlike the usually weak neck connections between spherical particles, the polyhedral La_2_Zr_2_O_7_ grains have strong conhesion through the face‐to‐face connection, resulting in better mechanical properties. In this work, *b *= 0.22 and *m *= 1 are obtained, suggesting much weaker dependence of the strength on porosity. It means that with the increase of porosity, high mechanical strength can be maintained (Figure 7e–i).^[^ ^36^ ^]^

### 3.2 Microstructure Design in Dense Nanocrystalline Ceramics

In nanoceramics, a marked increase in phase boundary and grain boundary volume fractions occurs as grain size diminishes to the nanometer scale. Under external forces, cracks readily propagate along the grain boundaries. The zigzag configuration of these boundaries deflects the path of crack propagation within nanocrystal grains, facilitating the absorption of additional fracture energy and thereby enhancing fracture toughness.^[^ ^37^, ^38^, ^39^ ^]^ Additionally, the homogenization of grains exerts a significant influence on the mechanical properties of nanoceramics.^[^ ^40^ ^]^ However, the presence of numerous noncoherent grain boundaries within the nanocrystal material diminishes the dislocation's holding capacity, promoting stress concentration and subsequently enhancing material strength while compromising toughness. Thus, the enhancement of ceramics' strength and toughness can be achieved through grain refinement, homogenization, and the strategic design of coherent/semi‐coherent interfaces.

Adjusting grain size is an important strategy to improve the mechanical properties of materials. In coarse grains, a substantial number of dislocations accumulate at the grain boundaries (with the quantity contingent on the distance between the dislocation source and the obstacle), leading to significant stress concentration. This scenario makes it easier to initiate dislocation sources in adjacent grains, thereby diminishing the yield strength. The basic idea of fine grain strengthening is to increase the number of grain boundaries by limiting the size of grains. Grain boundaries are the interfaces between adjacent grains, the existence of which can prevent crack propagation, thereby improving the toughness of ceramics. Under the external forces, in order to produce shear deformation in the adjacent grains, enough stress concentration must be generated at the grain boundary to make the dislocation move, which greatly improves the strength of ceramics. What's more, grain boundary segregation of impurity atoms can improve the yield strength and elastic modulus of nanomaterials effectively by decreasing the dislocation density of grain boundary, which makes the grain boundary structure more ordered and stable.^[^ ^41^ ^]^ Zhang et al. found that stable grain boundary networks interlocked with twin boundaries can effectively inhibit high‐temperature creep, which results from the structural relaxation of high‐density grain boundaries caused by plastic deformation in nanograined metals and alloys.^[^ ^42^ ^]^

**Figure 8:**
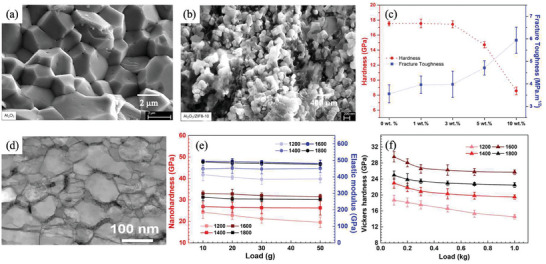
Fine grain strengthening. a) Pure Al_2_O_3_ and b) Al_2_O_3_/ZIF and c) Al_2_O_3_/ZIF showed remarkable improvement in fracture toughness. Reproduced with permission.^[^ ^43^ ^]^ Copyright 2023, American Chemical Society. d) High‐entropy carbide nanocrystal ceramics with ≈80 nm particle size. e) The nano hardness and elastic modulus and f) the Vickers hardness of high‐entropy carbide nanocrystal ceramics as a function of the applied load. Reproduced with permission.^[^ ^44^ ^]^ Copyright 2021, John Wiley and Sons.

The researchers prepared in situ MOF‐derived nanocarbon‐reinforced ceramics by carbonizing ZIF‐8‐derived carbon in alumina ceramics at high temperatures. As the content of ZIF‐8‐derived nanocarbon increases, the grain size of the final prepared composite becomes smaller and smaller (≈200 nm). This is because the nanocarbon accumulates at the grain boundaries, providing a pinning effect that inhibits grain growth. In addition, MOF‐derived carbon nanoparticles may act as a sliding transition layer to help dissipate energy during crack propagation, thereby improving the fracture toughness of the sample. Al_2_O_3_/ZIF‐8 showed a 67% improvement in fracture toughness compared to pure Al_2_O_3_ blocks (**Figure** **8**a–c).^[^ ^43^ ^]^ Chu et al. prepared high‐entropy carbide nanocrystal ceramics with a particle size of ≈80 nm by ultra‐high pressure sintering technology, with Vickers hardness up to 25.7 GPa and fracture toughness up to 4.3 MPa m^1/2^. Such excellent comprehensive mechanical properties are due to the fine grain strengthening effect of nanocrystal ceramics and a large number of grain boundary induced crack deflection, thus improving the toughness of the material (Figure 8d–f).^[^ ^44^ ^]^

**Figure 9:**
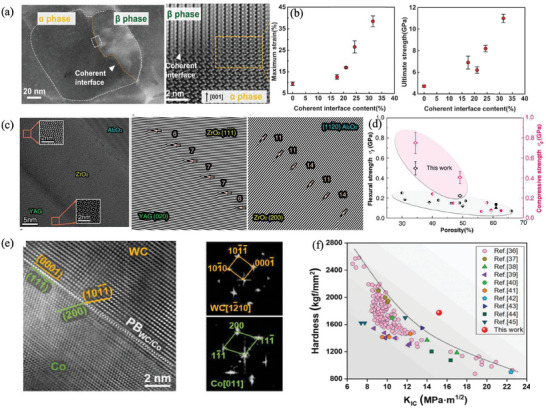
Coherent interface design to enhance interface strength. a) *α*‐phase and *β*‐phase biphase structure with Si_3_N_4_ of coherent interface and b) Maximum strain, ultimate strength versus coherent interface content. Reproduced with permission.^[^ ^45^ ^]^ Copyright 2022, The American Association for the Advancement of Science. c) Strong bonding interface of coherent/semi‐coherent between YAG/ZrO_2_/Al_2_O_3_ and d) Strength comparison with reported porous ceramics. Reproduced with permission.^[^ ^46^ ^]^ Copyright 2022, American Chemical Society. e) Coherent/semi‐coherent WC/Co phase boundary in nanoscale WC‐Co composites and f) Its hardness and fracture toughness compared with those of the counterparts reported in the literature. Reproduced with permission.^[^ ^47^ ^]^ Copyright 2023, Elsevier.

Coherent interface design is critical to enhancing interface strength. Coherent interfaces, characterized by minimal mismatches and the absence of pronounced distortions between adjacent crystals, facilitate the continuous transfer of stress and deformation across phase boundaries. In contrast, materials lacking interfacial coherence struggle to release stress concentration at the interface during deformation. This deficiency often results in unmitigated local deformation, creating a propensity for crack nucleation. Liu et al. were inspired by the bond‐switching mechanism in metal materials to achieve bond‐switching in covalently bonded ceramics by designing *α*‐phase and *β*‐phase biphase Si_3_N_4_ structures with coherent interfaces, resulting in macroscopic plastic deformation in covalently bonded ceramics. They observed the *β* to *α* phase transformation of Si_3_N_4_ at room temperature for the first time which was induced by the cumulative stress of bond conversion. Energy is dissipated through the phase transformation, releasing strain and avoiding fracturing failures. Si_3_N_4_ ceramics exhibit unprecedented compressive plastic deformation at room temperature, the shape variable is as high as 20%, and the compressive strength is increased to 2.3 times (≈11 GPa) (**Figure** **9**a,b).^[^ ^45^ ^]^ Su et al. used directional solidification to guide the formation of directional rod‐like pores and the directional growth of the ternary oxide eutectic phase, endowing the porous skeleton with extremely high density, and greatly improving the strength of the porous skeleton matrix. When the porosity is 34%, the flexural strength of the prepared porous composite ceramics is as high as 497 MPa at room temperature, setting a new record for the strength of porous ceramics at present. Thanks to the strong bonding interface of coherent/semi‐coherent, the specimen still has 324 MPa bending strength at 1773 K high‐temperature environment (Figure 9c,d).^[^ ^46^ ^]^ Song et al. formed a coherent/semi‐coherent WC/Co phase boundary in nanoscale WC‐Co composites, which promoted the uniform transfer of stress across the phase boundary between the hard phase and the ductile metal phase, and ensured the continuity of deformation between the metal phase and the ceramic phase, instead of forming stress concentration, leading to high hardness (1775 ± 23 kgf mm^−2^) and fracture toughness (15.20 ± 0.13 MPa m^1/2^), and the comprehensive mechanical properties achieved the highest level among those of the counterparts reported in the literature (Figure 9e,f).^[^ ^47^ ^]^

### 3.3 Toughened Ceramic Matrix Composites

Dispersing the reinforcement phase such as nanoparticles, whiskers/fibers in the ceramic matrix can produce a significant toughening effect.^[^ ^48^, ^49^, ^50^, ^51^, ^52^, ^53^ ^]^ The toughening mechanism can be explained as the following aspects:

Phase change toughening. The phase transformation consumes a lot of energy required for crack propagation, relaxes the stress at the crack tip, and prevents further crack propagation. At the same time, the volume expansion caused by the phase transformation causes the surrounding matrix to be compressed and cracks to close, thus improving the fracture toughness and strength. The most successful example is the ZrO_2_ martensitic phase transition. Chen et al. prepared Al_2_O_3_‐Mullite‐ZrO_2_‐SiC composites with bending strength and fracture toughness up to 970 MPa and 12.3 MPa m^1/2^, respectively. This is mainly due to the metastable tetramonal‐ZrO_2_ phase changing to monoclinic‐ZrO_2_ accompanied by 4–5% volume expansion and the number of *m*‐ZrO_2_ phases on the fracture surface increasing from 22% to 34%. The volume expansion generated by this phase transformation releases the driving force of crack tip expansion, thereby improving the fracture toughness of the material.^[^ ^54^ ^]^

**Figure 10:**
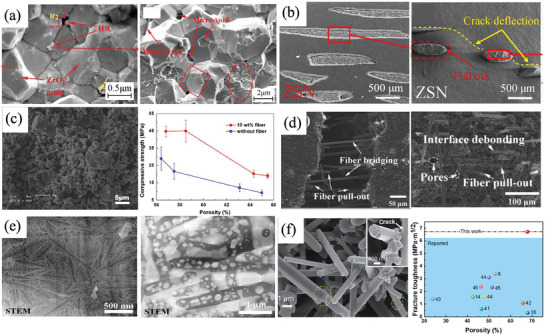
Bring in nanoparticles, whiskers/fibers, and other reinforcing phases in the ceramic matrix. a) Micro‐cracks caused by *t*‐ZrO_2_ to *m*‐ZrO_2_ phase transition. Reproduced with permission.^[^ ^55^ ^]^ Copyright 2018, Elsevier. b) ZrB_2_‐based ceramics containing Si_3_N_4_ short fiber (ZSN). Reproduced with permission.^[^ ^56^ ^]^ Copyright 2023, Elsevier. c) Fiber‐reinforced porous YSZ ceramics. Reproduced with permission.^[^ ^57^ ^]^ Copyright 2014, Elsevier. d) Short carbon fiber reinforced SiBCN ceramic. Reproduced with permission.^[^ ^58^ ^]^ Copyright 2023, John Wiley and Sons. e) Multiple toughening of zirconia‐alumina‐based ceramics. Reproduced with permission.^[^ ^59^ ^]^ Copyright 2022, John Wiley and Sons. f) SiC‐reinforced porous Si_3_N_4_ ceramics and its fracture toughness compared with literature. Reproduced with permission.^[^ ^30^ ^]^ Copyright 2022, Springer Nature.

Microcrack toughening. Within the interface of the ceramic matrix and the reinforced phase, variations in thermal expansion due to temperature fluctuations or volume disparities from phase transitions result in the formation of dispersed microcracks. As the primary fracture‐initiating crack propagates, these uniformly distributed microcracks play a crucial role in bifurcating the main crack. This process extends the path of main crack expansion, amplifies surface energy during the expansion phase, impedes swift crack propagation, and enhances the overall toughness of the material. Song et al. prepared HfB_2_‐ZrO_2_ matrix composite ceramics with bending strength of 1093.06 ± 24 MPa, fracture toughness of 8.17 ± 0.18 MPa m^1/2^ and Vickers hardness of 14.37 ± 0.21 GPa. Micro‐cracks caused by *t*‐ZrO_2_ to *m*‐ZrO_2_ phase transition contribute to improving the fracture toughness of the composites (**Figure** **10**a).^[^ ^55^ ^]^

Crack deflection and bridge toughening. Due to the obstructing effect of the reinforcing phase, the crack propagation path will change, thus lengthening the crack propagation path, reducing the stress concentration at the crack tip, and improving the fracture toughness of the ceramic. In addition, when cracks bridge, the compressive stress on the crack surface will offset part of the effect of external pressure, and prevent the further expansion of the crack.

Whisker/fiber pulling out and bridge toughening. When the crack encountered a high‐strength reinforcement phase on its expansion path, the columnar grain at the crack tip was forced to separate from the matrix and pulled out by external forces. The friction between the columnar crystal and the matrix can consume the energy of the applied load, so as to achieve the purpose of toughening. For example, the toughness of ZrB_2_‐based ceramics containing Si_3_N_4_ short fiber (ZSN) is 5.6 MPa m^1/2^, which is 20% higher than that of monolithic ZrB_2_ ceramics (4.7 MPa m^1/2^) (Figure 10b).^[^ ^56^ ^]^ The compressive strength of the porous YSZ ceramics was improved significantly with the introduction of fiber. Compared with the specimens without fiber addition, the compressive strength of fiber‐reinforced specimens was almost doubled and the highest reached 45.0 MPa with a porosity of 58.5% (Figure 10c).^[^ ^57^ ^]^

These toughening mechanisms often work together to reinforce the ceramic matrix composites. Li *et al.* prepared short carbon fiber reinforced SiBCN ceramic matrix composite, which significantly improved the fracture toughness of the composite under the synergistic action of crack deflection, interface debonding, fiber pull‐out, and other toughening mechanisms. The flexural strength reached 5.3 ± 2.1 MPa and the bending strength reached 44.0 ± 3.8 MPa (Figure 10d).^[^ ^58^ ^]^. The zirconia‐alumina‐based ceramics prepared by Zheng et al. can realize the synergistic toughening of multiple factors such as multistage nanostructure, columnar crystal, *t‐m* transformation of zirconia and crack deflection, and greatly improve the mechanical properties of the ceramics. The sample has extremely high hardness (HV20 GPa) and excellent toughness (16 MPa m^1/2^), and the bending strength reaches 1300 MPa (Figure 10e).^[^ ^59^ ^]^ Wang et al. prepared high‐porosity and high‐strength porous Si_3_N_4_ ceramics. They found that the carbon residue can form a strong SiC second phase which is evenly distributed in the lap of *β*‐Si_3_N_4_ grain and plays a role in napping grain boundaries. Crack deflection, bridge, and grain pulling out were observed in the fracture surface, leading to a maximum compressive strength of 47.75 MPa and fracture toughness of 6.71 MPa m^1/2^ with porosity of 67.83%, which is much higher than the value in literature (Figure 10f).^[^ ^30^ ^]^

### 3.4 Directional Structural Design

**Figure 11:**
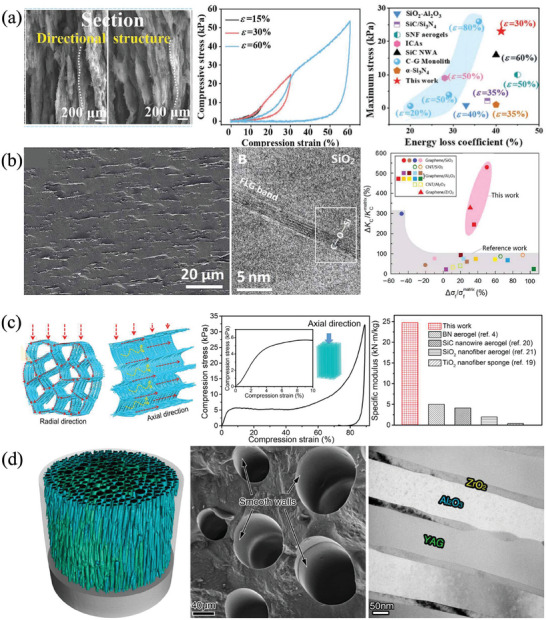
Directional structural design. a) Superadiabatic SiC aerogels prepared by an air suction effect induction strategy. Reproduced with permission.^[^ ^64^ ^]^ Copyright 2022, John Wiley and Sons. b) Directional 2D graphene in 3D ceramic substrates and comparison diagram with other reported ceramics reinforced with carbon nanostructures. Reproduced with permission.^[^ ^65^ ^]^ Copyright 2020, The American Association for the Advancement of Science. c) Honeycomb structure SiC@SiO_2_ by directional freezing pouring. Reproduced with permission.^[^ ^21^ ^]^ Copyright 2020, The American Association for the Advancement of Science. d) Directional growth of ternary eutectic phase construct by directional solidification. Reproduced with permission.^[^ ^46^ ^]^ Copyright 2022, American Chemical Society.

Ceramics usually appear isotropic due to their randomly oriented grains and intercrystalline phases. While the directional structural design makes ceramic exhibit unique and excellent properties along specific directions, which leads to anisotropy.^[^ ^60^, ^61^, ^62^ ^]^ For example, Wang et.al. found increasing the degree of whisker‐oriented alignment improved the resistance to damage of SiC/Si_3_N_4_ composites by the Vickers indentation method and R‐curve behavior.^[^ ^63^ ^]^ Cheng et al. prepared superadiabatic SiC aerogels by an air suction effect induction strategy which was used to subtly regulate the directional flow of SiO gas, inducing the directional growth and assembly of SiC nanofibers to form directional layered structures. In the axial direction, the lamellar structures were enables to withstand a high compressive stress (61.9 kPa) which is much higher than values in previously reported SiC materials. Moreover, the oriented layered SiC aerogel showed excellent large strain elasticity (60%) and fatigue resistance for 10 000 compressions (**Figure** **11**a).^[^ ^64^ ^]^ Wan et al. introduced directional 2D graphene into 3D ceramic substrates (alumina, silicon oxide, zirconia, etc.) to transform the catastrophic fracture failure mode of brittle ceramics into a stable crack propagation behavior, improving the mechanical toughness by 250–500% (Figure 11b).^[^ ^65^ ^]^ Pan et al. prepared a honeycomb structure SiC@SiO_2_ by directional freezing pouring, which obtained anisotropic mechanical properties and thermal conductivity. The thermal conductivity of transverse heat transfer is only 40% of that of longitudinal aerogels, where the longitudinal aerogels exhibit higher hardness and compression modulus (24.7 KN m kg^−1^) than other fiber‐assembled aerogels (Figure 11c).^[^ ^21^ ^]^ Su et al. used directional solidification instead of sintering to achieve the construction of a skeleton matrix. The rapid coupled directional growth of ternary eutectic phase is conducive to the formation of nanostructures and high‐density skeleton matrix, the flexural strength is as high as 497 MPa at room temperature when the porosity is 34%, which is the highest value for porous ceramics at present (Figure 11d).^[^ ^46^ ^]^ Ma et al. fabricated porous multilayered mullite ceramics with strong anisotropy properties via gel‐casting. The differences in compressive and flexural strengths in different directions were 14 (±0.7) and 5 (±0.2) MPa respectively, and the anisotropy factor of thermal conductivity was up to 0.98.^[^ ^66^ ^]^ Jia et al. reported a laminated SiC‐SiO*x* nanowire aerogel that exhibits mechanical robustness and flexibility, and good thermal insulation. It shows a high compressive stress of 1255 ± 116.3 kPa at 80% strain, high tensile stress of 399 ± 83.4 kPa, and high bending strength of 261 ± 11.4 kPa, which are several to tens of times higher than other resilient ceramic aerogels, showing improved load bearing capacity under diverse deformations.^[^ ^11^ ^]^

## 4 Integrating Machine Learning Assisted Simulations Toolbox

**Figure 12:**
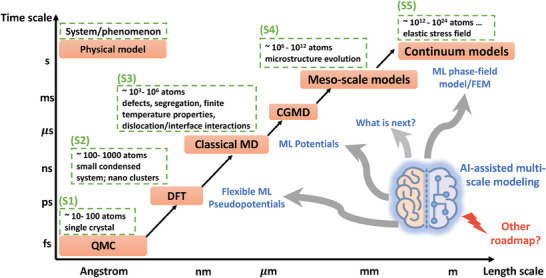
Multi‐scale materials modeling assisted by AI techniques. QMC stands for quantum Monte Carlo, DFT for density functional theory, CGMD for coarse‐grained molecular dynamics, and FEM for finite element method.

Experimental observations and advanced techniques have significantly enhanced our understanding of the structure‐property relationship in nanoceramics. Various types of nanoceramics have been developed with densities exceeding 90% and grain sizes below 100 nm. The advancements in computational capabilities and simulation methodologies have led to the increased importance of computer simulations in materials science, including the design of nanoceramics with advanced structural and functional properties. Density functional theory (DFT)^[^ ^67^ ^]^ calculations often provide accurate descriptions of material properties; however, due to the high computational cost and limited scalability (up to ≈1000 atoms), DFT is insufficient for investigating critical properties such as microstructure evolution in nanoceramics research. A prevalent approach in materials science is multi‐scale modeling^[^ ^68^ ^]^ in **Figure** **12** and the time and length scales can be extended to match experimental conditions. Specifically, at the nanometer scale and below, atomistic simulations^[^ ^69^ ^]^ can reveal fundamental structural and property information of nanoceramics, such as defect properties, phase transformations, and mechanical behavior. At the micrometer scale and larger, mesoscale simulations, such as the phase‐field (PF) model^[^ ^70^ ^]^ and finite element method (FEM),^[^ ^71^ ^]^ offer insights into microstructural evolution, including grain boundary (GB) development and resulting mechanical responses, which can be compared to experimental findings. Thermodynamic calculation techniques, such as CALPHAD,^[^ ^72^ ^]^ also play a vital role in the design of nanoceramics by calculating bulk and GB phase diagrams.^[^ ^73^ ^]^ In the past decade, rapid progress in artificial intelligence (AI) and machine learning (ML) techniques^[^ ^74^ ^]^ has led to their widespread application in addressing various scientific challenges within the “AI for Science”^[^ ^75^ ^]^ framework. As a subfield, “AI for Materials Science”^[^ ^76^ ^]^ has flourished, with AI emerging as a powerful tool for solving problems related to structural materials (such as alloys and ceramics) and new‐energy materials. This review aims to discuss the application of various simulation methods in nanoceramic research and explore how AI techniques can be integrated with these simulation methods to further advance the field.

### 4.1 Atomistic Simulations for Nanoceramics

Atomistic simulations play a crucial role in obtaining atomic‐level information and serve as the foundation for computer simulations in materials science. Two primary types of atomistic simulations exist: quantum mechanics‐based first‐principles methods and classical molecular dynamics (MD) or Monte Carlo (MC) simulations. DFT^[^ ^67^ ^]^ is a well‐known example of the first‐principles method, offering reliable and accurate results for a majority of material properties. However, DFT is computationally inefficient, with its speed typically scaling with the cubic power of atom numbers. The ongoing development of linear‐scaling DFT methods^[^ ^77^ ^]^ holds promise for addressing this efficiency issue. DFT is generally constrained by size scale (less than 1000 atoms) and time scale (less than 1 ns), making it unsuitable for many simulations involving phenomena such as defect interactions and solid‐state phase transformations. Several first‐principles calculation software packages, including VASP,^[^ ^78^ ^]^ ABACUS,^[^ ^79^ ^]^ and QUANTUM ESPRESSO,^[^ ^80^ ^]^ offer a friendly interface to perform DFT calculations for different materials. In contrast, classical MD/MC simulations are more computationally efficient than DFT, with speeds usually scaling linearly with the atom number. The reliability of classical MD/MC simulations primarily depends on the accuracy of the underlying interatomic potential, which describes atomic interactions between different atoms. Various types of interatomic potentials exist, including Lennard–Jones potential,^[^ ^81^ ^]^ ReaxFF reactive force field,^[^ ^82^ ^]^ Born–Mayer–Huggins,^[^ ^83^ ^]^ and Born–Meyer–Buckingham^[^ ^84^ ^]^ potentials for ceramics, as well as embedded‐atom method (EAM)^[^ ^85^ ^]^ and modified EAM (MEAM)^[^ ^86^ ^]^ for metals and alloys. These interatomic potentials have successfully contributed to understanding and providing physical insights into experimental observations. As demands for material properties and design have increased, classical interatomic potentials have proven to be unreliable in predicting certain structural and functional properties.^[^ ^87^, ^88^, ^89^, ^90^ ^]^ Consequently, there is an urgent need for the development of alternative approaches in atomistic simulations to address these challenges.

In recent years, ML methods have been employed in the development of interatomic potentials, resulting in the creation of various ML potentials (MLPs) for different material systems. These MLPs have significantly advanced the field of materials science, offering improved accuracy and computational efficiency. One of the earliest MLPs, the Behler‐Parrinello (BP) MLP,^[^ ^91^ ^]^ was developed in 2007 using a neural network framework. It characterizes atomic environments through atom‐centered symmetry functions, with the latest fourth‐generation BP‐MLP incorporating long‐range charge transfer capabilities.^[^ ^92^ ^]^ In 2010, the Gaussian approximation potential (GAP) method^[^ ^93^, ^94^ ^]^ was introduced, utilizing Gaussian Process Regression—a nonlinear tool closely related to linear and kernel ridge regression techniques. GAP considers atomic environments up to a cutoff distance, using a descriptor vector to represent the neighborhood. A kernel function is then applied to distinguish different atomic environments. More recently, the Deep Potential (DP) method was established in 2017^[^ ^95^ ^]^ and has been applied to study various properties of diverse materials.^[^ ^96^ ^]^ DP employs a neural network form and incorporates different atomic descriptors, ranging from two‐body and three‐body embeddings to hybrid descriptors. The open‐source DeepModeling community^[^ ^97^ ^]^ was founded based on DP, and the accuracy and computational efficiency of DP have been continuously improved over the past 5 years. In addition to the aforementioned MLPs, several other types of MLPs have been developed, such as spectral neighbor analysis potentials,^[^ ^98^ ^]^ moment tensor potentials,^[^ ^99^ ^]^ and SchNet.^[^ ^100^ ^]^ These advancements in MLPs are helping to push the boundaries of materials science and enable more accurate and efficient simulations and predictions.

Nanoceramics consist of various grains and grain boundaries (GBs). DFT is a reliable approach for calculating the fundamental structural and functional properties of single grains or crystals in these materials. Elastic constants can be determined from strain‐stress or strain‐energy relationships, where a series of strains are applied to the equilibrium structure, and the corresponding energies or stresses are measured. Based on these elastic constants, bulk, shear, Young's modulus, and Poisson's ratio can be calculated using Voigt–Reuss–Hill approximations and empirical equations.^[^ ^101^ ^]^ Among the mechanical properties of single crystals, dislocation properties are the primary carriers of plastic deformations. Recently, Li et al.^[^ ^102^ ^]^ utilized DFT calculations to determine the generalized stacking fault energy (GSFE)^[^ ^103^ ^]^ profiles for CsPbX_3_ (X = Cl, Br, or I) perovskites. They demonstrated that the {110}<1$\bar{1}$0> slip system has the lowest energy barrier, consistent with experimental observations. DFT was further employed to study electron localization functions and density of states in CsPbX_3_ to reveal the underlying physics of the GSFE profiles. For other fundamental properties, thermal conductivity can be derived from phonon profiles, which can be calculated using the Phonopy package^[^ ^104^ ^]^ and DFT for single crystals. Surrogate properties representing corrosion, such as surface energies, electron work functions, and hydrogen emission reaction rates, as well as functional properties like electronic and electrochemical properties,^[^ ^105^ ^]^ are readily obtainable from DFT calculations. Phase transformations, especially between solid‐solid phases, are sometimes challenging to access using DFT calculations due to limited size and time scales. In these cases, efficient molecular dynamics (MD) simulations can provide additional physical insights. Recently, VASP developers^[^ ^106^ ^]^ incorporated the training of MLPs into the software and generated an MLP for zirconia through on‐the‐fly training. The developed MLP can accurately describe the basic properties of cubic, tetragonal, and monoclinic phases and the phase transition temperatures between different phases, in comparison to experimental data.

**Figure 13:**
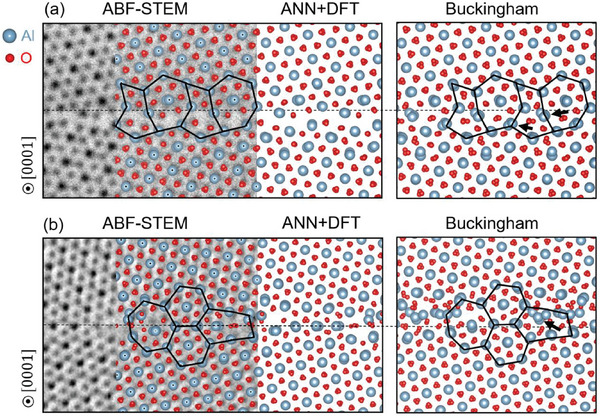
Comparison of the STEM images and the lowest‐energy structures predicted by ANN (BP‐MLP)/Buckingham interatomic potentials and subsequent DFT validation for a) the Σ7(4$\bar{5}$10) GB and b) the Σ31(7$\bar{11}$40) GB. Reproduced with permission.^[^ ^110^ ^]^ Copyright 2023, Elsevier.

**Figure 14:**
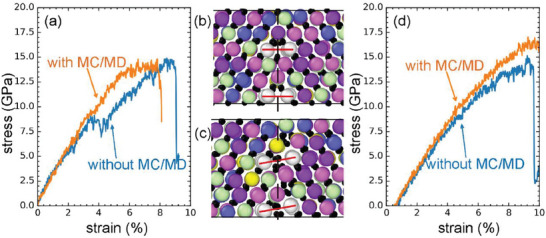
a) Stress‐strain curves of the Σ3(112) grain boundary with the initial structure being unsheared and b) unsheared c) sheared structures of the Σ3(112) grain boundary. d) Stress‐strain curves of the Σ3(112) grain boundary with the initial structure being sheared. The labels “with” and “without MC/MD” indicate whether MC or MD simulations were performed before the tensile simulations. In (b) and (c), the carbide is high entropy (Hf_0.2_Zr_0.2_Ta_0.2_Nb_0.2_Ti_0.2_)C, where small black spheres represent carbon atoms, and the large colorful spheres denote metal atoms (Ti: blue, Zr: yellow, Hf: pink, Nb: green, Ta: purple). Reproduced with permission.^[^ ^112^ ^]^ Copyright 2022, Elsevier.

In the case of polycrystalline nanoceramics, which consist of various grains and GBs, DFT calculations become infeasible due to low computational efficiency. MD serves as a more suitable tool for studying polycrystalline nanoceramics. For instance, Liang et al.^[^ ^107^ ^]^ employed MD simulations using the interatomic potential from Vashishta et al.^[^ ^108^ ^]^ to investigate the hot‐press sintering process of AlN nanoceramics. They specifically considered samples with varying nanoparticle sizes and hydrostatic pressures to elucidate their effects on the sintering process. In another study, Zhang et al.^[^ ^109^ ^]^ used MD simulations to analyze phase transformation behavior around GBs and the shape‐memory effect in yttria‐stabilized tetragonal zirconia bicrystals with different misorientations. Their results demonstrated that [011]/[01$\bar{1}$]‐oriented (parallel to the loading direction) bicrystals exhibit higher strength than the perpendicular ones and even surpass single crystals in strength. Understanding the structure‐property relationship of GBs is crucial for determining polycrystalline properties in nanoceramics like *α*‐Al_2_O_3_. Due to the limited flexibility of empirical interatomic potentials, these potentials can produce reasonable results for low‐Σ GBs but not for high‐Σ ones. To address this issue, Yokoi et al.^[^ ^110^ ^]^ trained a BP‐MLP based on DFT training datasets containing energies and atomic forces. **Figure** **13** shows the comparison between the STEM images and the lowest‐energy GB structures from BP‐MLP/Buckingham^[^ ^111^ ^]^ and DFT, where the potentials were used to screen different GB structures using a simulated annealing method followed by DFT single‐point calculations. When compared to STEM experimental results, the configurations predicted by BP‐MLP show good agreement while those by Buckingham potential are more disordered indicated by the black arrows in Figure 13 In other applications of MLPs, Dai et al.^[^ ^112^ ^]^ applied DP which was fitted from DFT calculations in MD to study the GB segregation of various metal carbides and analyze the effect of element segregation on GB strength. **Figure** **14** shows the tensile stress‐strain curve of one GB and the GB structure in (Hf_0.2_Zr_0.2_Ta_0.2_Nb_0.2_Ti_0.2_)C multi‐principal component carbides. The results show that GB structures undergo reconstruction under tension (different structures between Figure 14b,c) and higher GB strength can be obtained in metal carbides without this structural reconstruction (see Ref.[112]). These examples demonstrate how AI‐assisted atomistic simulations can enhance our understanding of GB properties in nanoceramics, providing valuable insights for the development and optimization of these materials.

With the emergence of large AI models like ChatGPT, large pretrain/foundation AI models have been recently developed for atomistic simulations in materials science^[^ ^113^, ^114^, ^115^, ^116^ ^]^ and have been applied in ceramic materials studies. Merchant et al.^[^ ^114^ ^]^ pretrained a general‐purpose MLP for bulk solids based on the first‐principles GNoME database covering diverse materials structures and elements. With the increase of the pretraining dataset, the accuracy of the pre‐trained MLP improves in classifying unseen compositions as inorganic superionic conductors, demonstrating the high transferability of the pretrained MLP. Zhang et al.^[^ ^115^ ^]^ pretrained a DPA‐2, a novel architecture for a large atomic model, on a dataset covering 73 elements in the periodic table. Then a distilled model for the solid‐state electrolyte Li_10_SnP_2_S_12_ was derived from a DPA‐2 model fine‐tuned with ≈1% of the previous training datasets of Li_10_SnP_2_S_12_. This distilled model shows satisfactory agreement with previous DPMD and DFT results on the diffusion constants of Lithium ions. Batatia et al.^[^ ^116^ ^]^ utilized the MACE architecture to train a general‐purpose foundation ML (MACE‐MP‐0) model on the Materials Project database^[^ ^117^ ^]^ containing 150 k inorganic crystals. This MACE‐MP‐0 model demonstrates good qualitative and, in some cases, quantitative accuracy for different material properties. One application of the MACE‐MP‐0 model in ceramic materials is studying defects and bulk diffusion in Al_2_O_3_. The diffusivities for Al from MACE‐MP‐0 model are within one order of magnitude compared to experimental values. For elemental defects such as Y and Co, the dopant atom migration minimum energy is accurate for Y but only qualitative for Co compared to DFT single‐point calculations. The development and fine‐tuning of large AI models for atomistic simulations of nanoceramic materials are a rapidly evolving field, with significant potential for multi‐component nanoceramic materials.

### 4.2 Meso‐Scale and CALPHAD Simulations for Nanoceramics

Meso‐scale simulations, such as PF and FEM modeling, play a vital role in understanding the properties of nanoceramics, including microstructure (porosity, grain size, texture, et al.) evolution, which are integral areas of focus in ceramic research. These simulations require input data on relevant defect properties, including point defect formability and diffusivity, dislocation gliding barriers, and GB energy and mobility. These inputs can be obtained either from experiments or atomistic simulations. For BaTiO_3_‐related ferroelectric nanoceramics, Su et al.^[^ ^118^ ^]^ utilized PF simulations based on the time‐dependent Ginzburg–Landau kinetic equation to study the intrinsic and extrinsic effects of GB on physical properties, with grain sizes ranging from 10 to 170 nm. The intrinsic part is associated with the local property of the GB, while the extrinsic part is attributed to domain structural dynamics. The PF modeling results revealed that the extrinsic effect dominates when the grain size is above 50 nm, while the intrinsic effect becomes dominant when the grain size is reduced below 50 nm for poled BaTiO_3_ nanoceramics. This transition from extrinsic to intrinsic dominance mirrors the shift from low‐frequency to high‐frequency loading. Cai et al.^[^ ^119^ ^]^ employed PF modeling to investigate the dielectric breakdown strength of ferroelectric nanoceramics (BaTiO_3_‐related) and FEM to study the ferroelectric hysteresis loop. Their findings indicated that dielectric strength increases, and dielectric permittivity decreases with decreasing grain size. Similarly, a FEM model based on a modified hyperbolic tangent model was applied to examine the synergy effect of breakdown strength and polarization in nanograined BaTiO_3_‐based ceramics, verifying an optimal grain size of ≈70 nm.^[^ ^120^ ^]^ For ZnO piezoelectric ceramics, Kim et al.^[^ ^121^ ^]^ used COMSOL software^[^ ^122^ ^]^ to perform FEM simulations on 3D hollow nanostructures and confirmed an extended elastic limit compared to fully dense ZnO. The 3D hollow nanostructured ZnO not only exhibits enhanced elastic strain limits but also maintains a piezoelectric coefficient similar to single crystals. For other ceramic materials, Gong et al.^[^ ^123^ ^]^ utilized ABAQUS commercial software^[^ ^124^ ^]^ for FEM modeling to investigate the effects of grain size (5 µm–70 nm), GB fracture energy, and defects on the mechanical properties of alumina. The results demonstrated that grain size and GB are interrelated, and mechanical properties can be improved by reducing grain size and GB density while increasing GB fracture energy. Wang et al.^[^ ^125^ ^]^ established a microstructure evolution model based on cellular automata for Al_2_O_3_/TiC/TiN micro‐nano‐composite ceramic tool materials, aiming to optimize the sintering parameters and design ceramics with enhanced mechanical properties. Simulation results indicated that ceramics with a uniform microstructure and excellent overall performance could be achieved with a sintering temperature of 1650 °C and a holding time of 15 min. Yu et al.^[^ ^126^ ^]^ employed PF simulations to study the nanoprecipitation mechanism and microstructure evolution of Al_2_O_3_/ZrO_2_ supersaturated solid solution micro‐powders (AZ‐SSP) during their fabrication into Al_2_O_3_/ZrO_2_ nanocomposite ceramics. They analyzed three AZ‐SSP samples with different Al_2_O_3_ molar ratios, revealing that two exhibited spherical and elongated ZrO_2_ particles, while the third displayed a continuous interlocking structure, all consistent with experimental microstructures. To further increase the computational efficiency of PF modeling and expedite the exploration of the process design space, Yabansu et al.^[^ ^127^ ^]^ successfully established reduced‐order process‐structure evolution linkages for forecasting grain and pore size distributions during the sintering of porous ceramics, calibrated with PF results. Specifically, they utilized ceramic grain and pore chord length distributions to quantify microstructure, implemented Principal Component Analysis to obtain a high‐fidelity low‐dimensional representation of microstructure statistics, and developed local Gaussian Process ML models in conjunction with autoregressive time series to construct reduced‐order models. The high accuracy of the reduced‐order models holds the potential for addressing inverse problems in nanoceramics process design and facilitating multi‐scale materials simulation through highly efficient computation algorithms. These studies highlight the importance of mesoscale simulations, such as PF and FEM modeling, assisted by ML and data science techniques, in advancing our understanding of nanoceramic properties and guiding the development of optimized materials.

CALPHAD,^[^ ^72^ ^]^ which stands for “CALculation of PHAse Diagrams,” is a computational approach that has enabled the design of various materials by modeling thermodynamics and atomic dynamic properties. The recently developed zentropy theory, which combines DFT and statistical mechanics, enhances the precision of free energy calculations and phase transition temperature predictions.^[^ ^72^ ^]^ Recently, Pang et al.^[^ ^128^ ^]^ combined a lattice engineering approach, CALPHAD, and supervised ML tools to screen zirconia compositions for shape‐memory (SM) zirconia ceramics design. Four surrogate properties were considered for the design of zirconia: i) commensurate interfaces between the transforming phases (λ_2_), ii) transformation volume change (Δ*V*/*V*), iii) solid solubility, and iv) martensite starting temperature (*M_s_*). Figure 1 of Ref.[108] illustrates the modeling workflow to design SM zirconia‐based ceramics. The chemical composition is first input into a CALPHAD model to predict solubility and transformation temperatures (*T*_0_ is obtained from the experiment and *M_S_* is derived from CALPHAD). Simultaneously, the chemical composition is input into an ML model to compute the lattice parameters of different zirconia phases at the transformation temperature. The predicted lattice parameters are then passed to a crystal kinematic compatibility model to calculate the remaining two surrogate properties, λ_2_ and Δ*V*/*V*. Finally, the results of four surrogate properties are combined to make reliable predictions of SM zirconia with a small hysteresis temperature for new compositions. Polycrystalline ZrO_2_‐17TiO_2_‐3AlO_1.5_‐6CrO_1.5_ (with mole percent of TiO_2_, AlO_1.5_, and CrO_1.5_ are 17%, 3%, and 6%, respectively) was fabricated experimentally, and an extremely low hysteresis temperature of 15 K was measured. This example demonstrates how thermodynamic calculations and ML methods can work together to enable efficient nanoceramics design.

### 4.3 Integration of Machine Learning

In the field of computational simulation and design of nano ceramics, AI is a powerful tool that is expected to play an increasingly significant role. To achieve this, several objectives need to be accomplished. First, the development of an AI‐native nanoceramics design platform based on Figure 1 of Ref.[108] can establish a new paradigm for nanoceramics design. Although Materials Studio^[^ ^129^ ^]^ and Virtual Lab^[^ ^130^ ^]^ currently facilitate atomistic simulations of material properties, a seamless integration of AI techniques and high‐throughput calculations across different time and size scales is lacking. In addition to the existing components, such as CALPHAD and ML tools in Figure 1 of Ref.[108], the AI‐native platform could incorporate methods like DFT calculations with flexible pseudopotentials,^[^ ^131^ ^]^ automatic workflows for developing, fine‐tuning and testing^[^ ^132^ ^]^ MLPs like DP and pre‐trained DPA‐2 models, ML‐assisted mesoscale modeling like JAX‐FEM^[^ ^133^ ^]^ for ML‐aided computational mechanics, and generative AI models^[^ ^134^ ^]^ for inverse design. These rapidly developing methods present challenges in effective integration and quantifying error propagation, which need to be addressed consistently. Second, there exists a gap between experimental and computational materials science, necessitating the enhancement of practical applications and predictive capabilities of AI‐assisted simulations. In multi‐scale modeling, as previously mentioned, data transfer and error propagation^[^ ^68^ ^]^ remains a key issue and is currently an active area of investigation. Alternatively, the rapid development of large language models (LLMs) presents another method or roadmap in Figure 12 for bridging the gap between experiments and simulations. LLMs can be viewed as encyclopedias trained on extensive corpora, encompassing many materials science domains. Data obtained from multi‐scale modeling can be used to fine‐tune the LLM, creating a specialized LLM for nanoceramics design. This specialized LLM can then offer guidance on optimal compositions and experimental conditions in response to input questions. Another strategy involves prompt‐engineering the LLM to distill nanoceramics knowledge database^[^ ^135^ ^]^ and integrate this database with those from multi‐scale modeling. By consolidating databases from both experimental and simulation sources, addressing data inconsistencies, and training AI models on this combined dataset, the predictive power of the models can be enhanced for practical applications. Third, the establishment of open‐source communities is crucial for enhancing software development and reducing time and monetary waste. Addressing issues related to proper acknowledgment and intellectual property is essential for the growth of open‐source communities. Lastly, increased interaction between simulation researchers and experimentalists is necessary to identify specific problems and solve more complex issues. With the foundation of an AI‐native platform, LLM knowledge database, and open‐source communities, the simulation aspect of nanoceramics research can expand horizontally, providing a fundamental basis for new platforms and communities, as well as vertically, enabling the resolution of diverse problems in the field.

## 5 Summary and Outlook

Nanoceramics, characterized by microstructures within the nanometer scale, represent an emerging class of ceramics. As the nanoceramics family diversifies and progresses toward multifunctionality and diverse applications, intrinsic brittleness emerges as a significant impediment to widespread implementation. Consequently, this review prioritizes the exploration of damage‐tolerant nanostructure ceramics, delving into recent advancements that have sought to enhance the mechanical properties of nanoceramics. Noteworthy strategies encompass the preparation of elastic nano‐fibrous ceramic aerogels and the development of high‐strength load‐bearing ceramics. Moreover, the integration of machine learning‐assisted simulations and various simulation methods into nanoceramics research has unveiled substantial potential across numerous fields. The ensuing discussion outlines future directions for the development of nanoceramics:

1. Advancement of multi‐functional performance: A key avenue for future research involves enhancing the multi‐functional performance of nanoceramics. By amalgamating diverse nanomaterials, such as nanoparticles and nanotubes, and incorporating functional surface modifications, a comprehensive fine‐tuning of thermal, electrical, optical, acoustic, and other properties can be achieved. This approach renders the material more adaptable for applications across various fields, thereby expanding its potential in diverse scenarios.
2. Enhancement of mechanical properties: A future research focus should prioritize the refinement of mechanical properties in nanoceramics. Achieving high strength often necessitates increased matrix density and reduced porosity, which runs counter to the requirements for a flexible and elastic structure. Consequently, future developments must navigate the challenge of reconciling the apparent contradiction between strength and elasticity, ultimately producing nanoceramics with both exceptional strength and flexibility.
3. Advancements in large‐scale preparation Technology: A pivotal trajectory in nanoceramics research involves the low‐cost large‐scale preparation of homogeneous materials. Presently, laboratory preparation methods often face scalability constraints, resulting in uneven distribution of nanoparticle sizes. Future investigations should prioritize the development of more economical and efficient preparation techniques capable of achieving precise size control. This imperative will address the burgeoning demand for the large‐scale production of high‐quality nanoceramics for practical applications.
4. Environmental consciousness and sustainable practices: In the realm of nanoceramics preparation, heightened emphasis on environmental friendliness and sustainability is warranted. Researchers are encouraged to explore greener preparation methods, utilize degradable raw materials, and pioneer recycling technologies. This strategic shift toward eco‐friendly practices constitutes a fundamental direction for future developments in nanoceramics research.
5. International cooperation and standard‐setting: Since nanoceramics is an emerging field, international cooperation and standard‐setting will play a key role in its industrialization and commercialization. Through international cooperation, can jointly solve the preparation technology, performance testing, standards, and other aspects of the difficult problems, to promote the common development of this field.

In general, nanoceramics have great development potential, and future research will make greater breakthroughs in improving performance, expanding application fields, improving preparation technology, and realizing environmental friendliness and sustainability.

## Conflict of Interest

The authors declare no conflict of interest.
